# Supplementary figures and images for: Respiratory kinematics and the regulation of subglottic pressure for phonation of pitch jumps – a dynamic MRI study
Source: PLoS One. 2020 Dec 31;15(12):e0244539. doi: 10.1371/journal.pone.0244539 (PMC7775092; doi:10.1371/journal.pone.0244539)

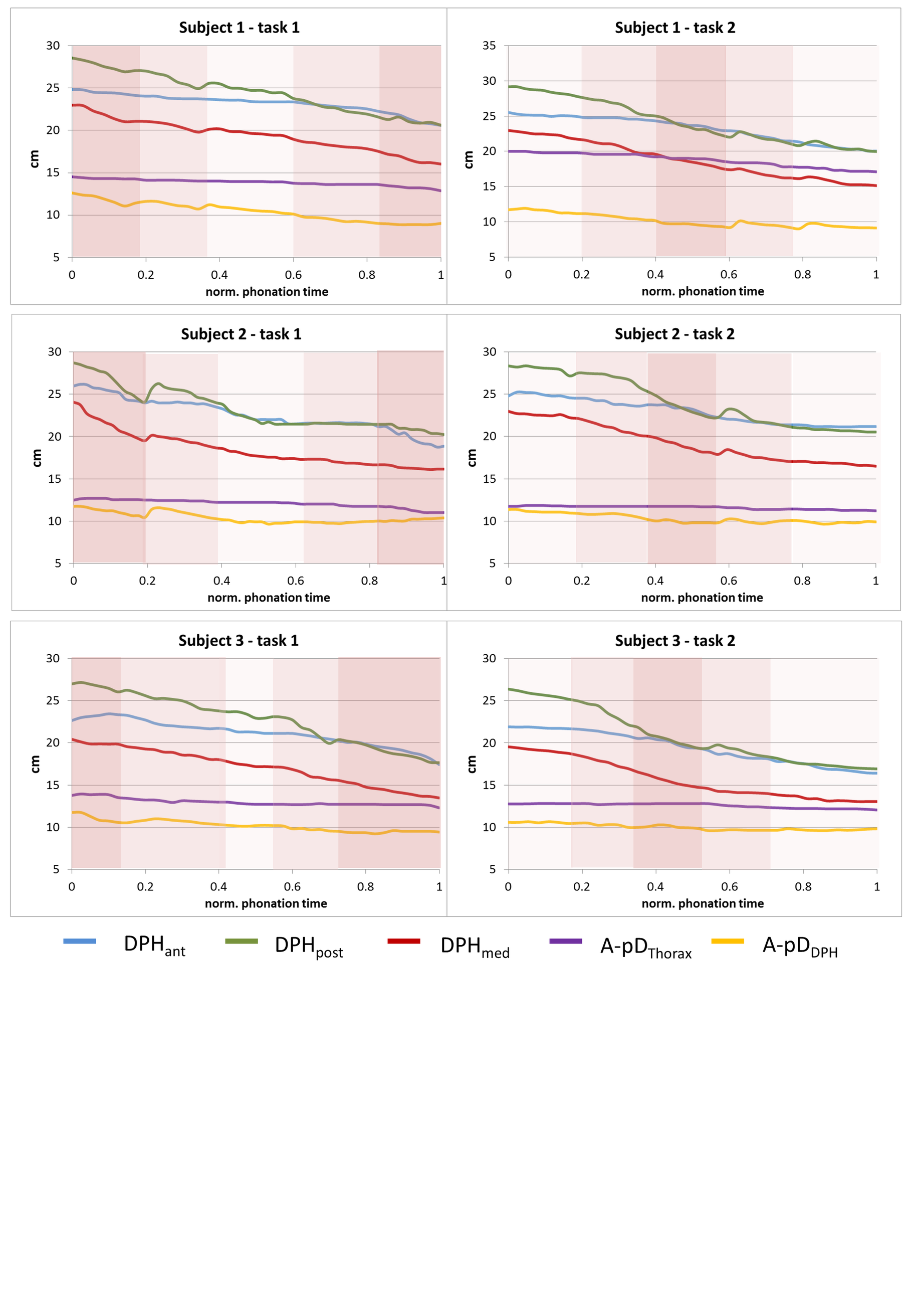

Supplement: S1 Fig — Different fos are marked with different shade taps indicating the jumps (darker shape higher fo and lighter lower fo). (TIF) [file pone.0244539.s001.tif]

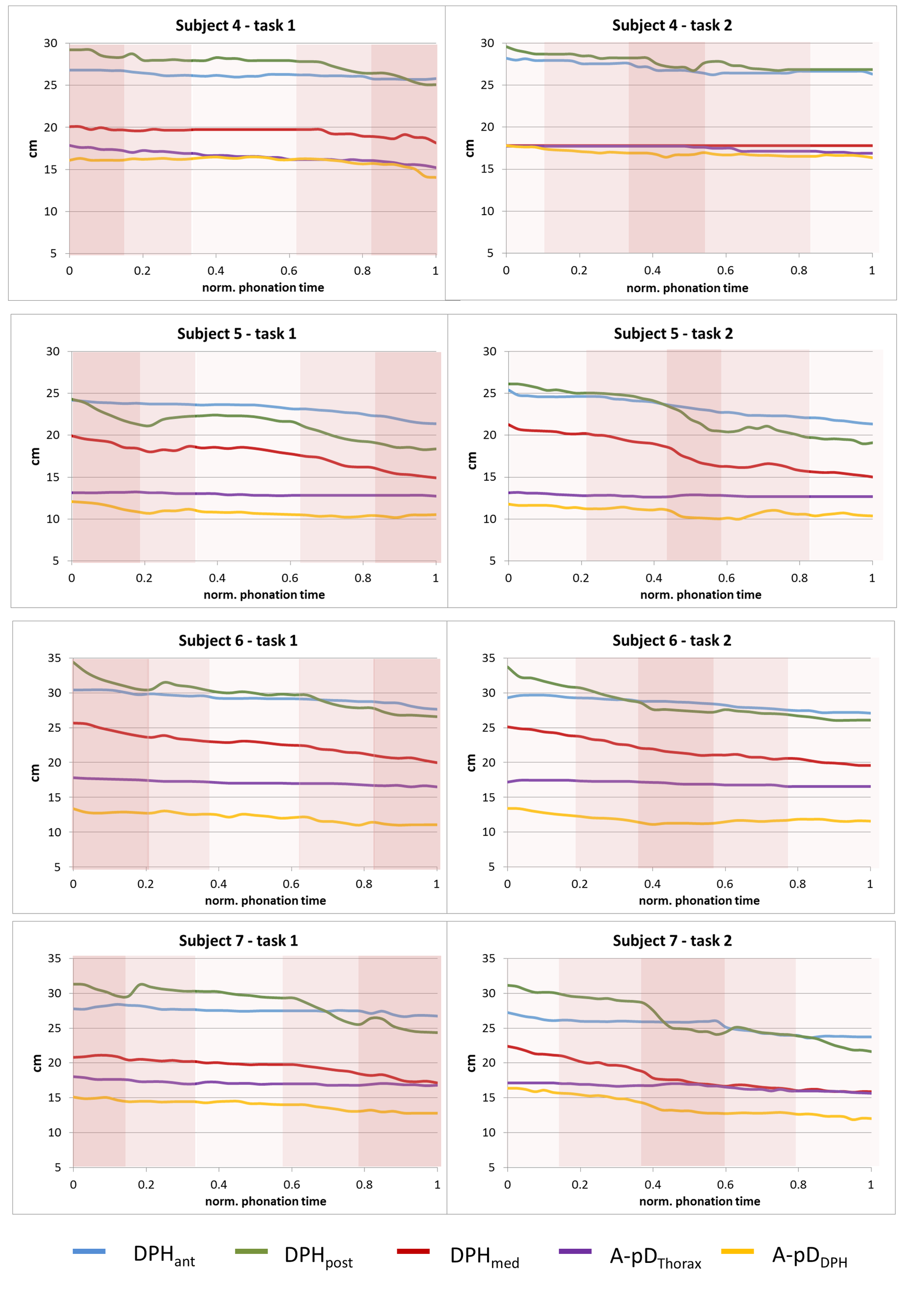

Supplement: S2 Fig — Different fos are marked with different shade taps indicating the jumps (darker shape higher fo and lighter lower fo). (TIF) [file pone.0244539.s002.tif]
