# Supplementary material for: Respiratory kinematics and the regulation of subglottic pressure for phonation of pitch jumps – a dynamic MRI study
Source: PLoS One. 2020 Dec 31;15(12):e0244539. doi: 10.1371/journal.pone.0244539 (PMC7775092; doi:10.1371/journal.pone.0244539)
Supplement: S1 Table — Significant differences (p < .05) are marked with darker colors. (DOCX) [file pone.0244539.s003.docx]

|  | DPH_ant_ | DPH_med_ | DPH_post_ | apD_Thorax_ | apD_DPH_ | DPH_right_ | DPH_left_ |
| --- | --- | --- | --- | --- | --- | --- | --- |
| DPH_ant_ |  | .93 | .99 | .01 | .64 | .96 | .95 |
| DPH_med_ | .63 |  | 1.00 | <.00 | .09 | 1.00 | 1.00 |
| DPH_post_ | .03 | .75 |  | .01 | .34 | .99 | .99 |
| apD_Thorax_ | .99 | .35 | .01 |  | .56 | <.00 | <.00 |
| apD_DPH_ | .00 | .02 | .59 | .00 |  | .12 | .11 |
| DPH_right_ | .98 | .98 | .24 | .85 | .001 |  | 1.00 |
| DPH_left_ | .99 | .95 | .16 | .93 | .001 | 1.00 |  |

S1 Table: p-values for differences in m_mean_ at different location for jumps upwards (red boxes) and m_max_ for jumps downwards (blue boxes) Significant differences (p<.05) are marked with darker colors.
