# Supplementary material for: Respiratory kinematics and the regulation of subglottic pressure for phonation of pitch jumps – a dynamic MRI study
Source: PLoS One. 2020 Dec 31;15(12):e0244539. doi: 10.1371/journal.pone.0244539 (PMC7775092; doi:10.1371/journal.pone.0244539)
Supplement: Attachment — Submitted filename: Rebuttal letter_final.docx [file pone.0244539.s006.docx]

**We thank the reviewers for their constructive critique and the helpful comments. In the following, we are trying to answer all reviewer questions individually**

# Reviewer #1

R.1.1: The authors refer to a pilot experiment that has previously been published using a similar technique on an overlapping sample of participants. It is important that the intro­duction clearly inform the readers on the difference between these two studies and the degree to which the present study is a novel extension versus a replication of previous findings. Though replications are very valuable I am concerned about the fact that the same participants were used in both experiments and that the authors may have sliced their data too thinly.

*More detailed information about the pilot study is now added to the introduction and discussion sections. The pilot study is presenting a proof-of-principle in dynamic imaging of the respiratory system during phonation (Traser et al., 2017). In this study for the first-time dynamic MRI was combined with EGG measurements during an experiment with professional singers. The singers phonated sustained at different pitches and loudness conditions. Anatomic measures were introduced and phonatory movement patterns were compared to respiratory. The pilot study solely investigated respiratory movements during sustained phonation. This deviates substantially from the current study where a detailed evaluation of subglottic pressure control for pitch jump phonation is presented. Here a new workflow was implemented, which included the extraction of the anatomic measures in a jump-time-window to enable an inter-individual comparison of movements of the respiratory system. Additionally this study was augmented with subglottic pressure measures in a second part of the experiment. Some of the subjects from the pilot study agreed to be also part of this study but there is no overlap in the presented data.*

R1.2: From visual inspection of the data the authors have decided to analyze the maximum change in distance between landmarks for some conditions (figure 8) or the mean change is distance between landmarks in others (figure 9). Since this choice was made post-hoc the authors have implicitly performed all pairwise tests within both measures, and should correct for them. Whereas present they appear not to perform any corrections for multiple tests. All analyses for both of these figures should be corrected for the ~40 separate tests that the authors have conducted (more if they have also visually inspected other possible measures such as the minimum distance). Stats for all of these tests should also be reported explicitly in a table.

*The decision for analysation of the maximum or mean change was based on the repeated measures ANOVA concerning changes of respiratory movements according to jump direction and the visual analysis of individual curves. A univariate ANOVA was then used to check for difference in mean or maximum movements and significant differences were then further analysed via post-hoc Turkey-HSD test. More detailed information on this analysis is now included in the method section, and we introduced the results of all post-hoc Turkey-HSD as a supplementary table including all p-values as suggested (supplement table 1). Standard error was included in the graphs instead of standard deviation.*

R1.3: Throughout the results section the authors describe the findings in terms of contrasts (in such a condition X measure Y was lower than in condition Z). However, they report statistical tests that appear to be omnibus anovas, which do not permit this interpretation. Please provide more careful interpretations of the statistics that are report or choose statistical tests that are more closely aligned with the inferences that interest the authors.

*Indeed, this was not sufficiently clarified in our method section and is now augmented: We performed repeated-measures ANOVA (factor 8 = gradient 1-8 in jump-time-window) in regards to differences in jump direction, jump time and concerning differences in location. We also performed univariate ANOVA when only one measure was analyzed like in mmax ode mmean. Posthoc analysis was then performed via Turkey HSD. The result section was adapted as recommended.*

R1.4: The authors should also report how they have handled non-independence in their data. Each task contained multiple pitch jumps of the same type, and it would be inappropriate to treat these replicate jumps as independent when they come from the same singer. It would be appropriate to use statistical methodology that can cope with this, such as linear mixed models, or to simply average across replicate jumps. From the very large degrees of freedom reported for the omnibus anovas, I suspect that this was not done. Even so I am confused by how large the degrees of freedom is here and would appreciate some clarification of what went into this analysis.

*More detailed information is now introduced in the results and methods section to clarify how non-independence was handled: Indeed, it is not appropriate to treat these replicate jumps as independent when they come from the same singer. We did not perform multiple performances of one task. For every task only the best one (best correspondence with the task in the opinion of the singer and the examiners) was chosen for analysation. The repeated measures ANOVA include the gradient changes of respiratory movement curves in 20% steps. This is a typical approach, which was also used in former studies in this context (Traser et al., 2017). In order to account for the factor that different jumps of the same subject were included in the statistical evaluation in the repeated measures ANOVA gender and subject were here regarded as covariates. Additionally to account for the factor that also different locations were included, we now also introduced the locations as covariate. This changes the value of the static parameters but not their significance.*

*The degrees of freedom are indicated and calculated in this way F(df of the effect of the model, df for the residuals of the model), e.g. with degrees of freedom of the effect of the model being 7 for the gradient in jump-time-window with 8 conditions (7 = 8-1) and degrees of freedom for the residuals of the model being 381 with 392 as the total sample size, 8 as the conditions and 3 as the number of predictors, covariates included (381 = 392 – 8 – 2 – 1).*

R1.5: Please provide the underlying data as per plos1 policy. Currently, only a subset of the data are provided and only for one participant. It would be most appropriate to include the raw MRI images organized in some sensible directory tree as well as all derived data.

*We agree and we support PLOS open data policy. Therefore we included in the supplement a figure with all jumps of all subjects as graph. Additionally we now also included a set of all Excel data for all jumps and all subjects as supplement (supplement data table). Raw MRI with sound is provided exemplary for one subject who agreed with the publication. The explicit publication of the data would only make sense with the corresponding audio recording (without audio pitch jumps are not recognisable). But with audio, this would interfere with the participant’s right to privacy. According to the PLOS Open Date guidelines for studies involving human participants, data must be handled so as to not compromise study participants' privacy. As the individual voice is recognisable (and all singers are pro­fessionally trained singers) is not possible to bypass this by de-identification or blocking portions of the database. Additionally, according to the PLOS open data policy „authors do not need to submit the raw data collected during an investigation if the standard in the field is to share data that have been processed.“ It is common in the field of dynamic MRI measurements for questions in voice physiology to provide exemplary MRI data, in which the quality of the images can be comprehended but not all single MRI images e.g.* (Echternach, Burk, Burdumy, Traser, & Richter, 2016a)*.*

## Minor concerns

abs) There should be no line breaks in the abstract *🡪 done*
abs) References to theory should come early in the abstract not late🡪 The abstract was adapted.
intr) The biomechanical review at the beginning of the introduction would benefit from an explanatory figure for non-specialists *🡪 we thank the reviewer for this suggest and included a new figure 1 which summarises the interaction of subglottic pressure and fundamental frequency*.

l53) the previous ultrasound study should be described in more detail, as with the pilot study 🡪 *done*
l93) "permanent" may not be the correct word 🡪 *changed to constant*
l118) please report this phoneme in IPA format🡪 *done*
l153) extra period *-> erased*
l163) what where the instructions for the out of scanner recordings? Should we be concerned about gross differences in SPL or Psub if singers were directing themselves towards a person or microphone that was farther away in the out of scanner test versus a microphone that is nearby in the scanner as well as the sense of being alone that one has in that context?

- *The singers were instructed to use the same SPL in both experimental parts (MRI and out of MRI). Additionally, both parts were executed in supine position and the distance to the microphone was similar and defined by a microphone stand. The manuscript was augmented in these points. Professional singers are used to control their vocal output also in very noisy surroundings (e.g. in an opera choir) but still a possible Lombard effect can not be totally excluded which could have made a difference between in MRI and out of MRI measurements. This limitation is now included in the discussion section.*

l199) remove the % sign from the equation *🡪 done*
l217) Something doesn't seem right in the denominator. Should you really be dividing by a larger number as you step through frames? *🡪 There was a mistake in the formula that is now corrected*
l226) if the threshold was p = 0.05 then do not report p < 0.01 in figures. P-values are never a measure of effect magnitude. The same applies to subsequent figures. *🡪Differentiation between p < 0.001/<0.05 was removed from the figure.*

Fig4) remove interpolation between points as you probably don't mean to imply that there are smooth transitions as depicted

*🡪 we thank the reviewer for this comment. The data shown was indeed measured in stable parts of the subject’s phonation and not continuously during the jump. Still we find the readability of the graph much improved by presenting the interpolation, as without the* *tracing of individual points would be very challenging. To avoid confusion, a note was included in the figure capture that clearly defines that the figures shows measures from a sustained period.*
l252) correct several typos in this section🡪 done
Figs 6 & 7) The means in these figures are not interpretable without error bars*.*

*🡪 We thank the reviewer for the very good suggestion and included standard errors in fig 6 as well as for the subglottic pressure, sound pressure level and open quotient deltas in figure 7. For curves in figure 7 error bars reduced the readability a lot. Therefore individual curves are displayed in the supplement figure 1 and 2 to show the variance across subjects.*

Figure 9)
l313) why have the authors switched from reporting effect sizes in eta squared to cohen's d? Is this intentional or a typographical error? Also, these effect sizes appear larger than I would expect for non-significant effects with the degrees of freedom report here. Please double check these values.

- *We thank the reviewer very much for this comment and changed all effect sized to eta squared as not to not confuse the reader. Values were corrected correspondingly.*

l243) adaption is not an english word, please revise here and throughout the discussion.

- *changed to adaptation in the whole manuscript*

l343) remove extra space *🡪 done*
l408) remove the (= note) notation here and throughout *🡪 = was removed*
l449) please clarify what you mean by rotatable MRI device *🡪 done*
l472) please remove the // notation and provide full sentences *🡪 done*

# Reviewer #2

R2.1: Line 32-33, and Line 337. "elevated" and "reduced" need to be referenced to some baseline. Elevated and reduced compared to what?*🡪 done*
R2.2: Line 37. "rip cage" should be "rib cage". 🡪 *Done in the whole manuscript*
R2.3: Line 38. "Diaphragm elevation primarily generated the force to increase subglottic pressure", but the diaphragm is an inspiratory muscle, and its elevation does not generate the force, rather, it is probably abdominal muscles that generate the force and elevate the diaphragm. *🡪 this passage was rewritten*
R2.4. Line 40. "underlines" should be "underline".*🡪done*
R2.5. Lines 84-96. I think the hypotheses could be highlighted more clearly, perhaps using a list format rather than a paragraph format. They should also be brought back to mind in the Results section.*🡪 the hypotheses were closer defined and an additional table was included with a concise summary in the introduction. This served as a base for the results section – here results were shown in the same order.*
R2.6. Line 102. I'm not sure it is a valid assumption that professional singers would be "less distracted by the noise".*🡪this passage was rewritten. Singers are used to auditory masking like in choir singing.*
R2.7. Lines 106-107. There is no explanation of how the VC and FEV1 measures were obtained. The VC measures in Table 1 are quite large - is it accurate? *🡪 the manuscript was augmented with details to the spirometer. Measures were obtained in an established clinical setup and should be correct.*
R2.8. Line 113. The pilot study has a very similar title. How is it different from the present study, beyond the addition of 2 subjects? *🡪 this difference is now explained more comprehensively in the introduction section. In the pilot study only sustained phonation was analysed.* Please confer to R1.1 for further clarification.
R2.9. Line 119. "with no pitch repetitions". What does this mean? *🡪 The pitch jumps were sung legato without separation between two pichtes. Manuscript was amended*
R2.10. Line 123 and Lines 127-135. Is the method employed here rtMRI or is it truly real time MRI, since the notes are held for so long? *🡪 yes, as the temporal resolution is 3 frames per second, this is a dynamic sequence.* (The image reconstruction was also performed real time.)
R2.11. Line 139 and Line 140. "coupola" should be "cupola".*🡪 done*
R2.12. Line 150. "detection of glottal opening (at 3/7)". What is "(at 3/7)" mean? Glottal opening was detected at 3/7 of the current cycle's amplitude *🡪 manuscript was amended to make this point clearer.*
R2.13. Lines 152-153. "estimated for each task ... from a steady state portion of each pitch." I found this sentence a bit awkward. There is an additional period "." at the end. *🡪 sentence was revised and second period was erased.*
R2.14. Line 170. "Rothenberg mask". It's not clear to me how this was used. *🡪a Rothenberg mask is a a circumferentially vented pneuotachograph mask with defined openings for a pressure intraorally placed pressure transducer. Section was augmented*
R2.15. Line 202. "EGG-MRI signal". What does this mean? EGG signal obtained simultaneously during MRI *🡪 section revised.*
R2.16. Lines 235-246. It's not clear where the degrees of freedom of "391" and "69" are coming from in the statistical analyses. *🡪*

*We corrected the degrees of freedom: The degrees of freedom are now indicated and calculated in this way F(df of the effect of the model, df for the residuals of the model), e.g. with degrees of freedom of the effect of the model being 7 for the gradient in jump-time-window with 8 conditions (7 = 8-1) and degrees of freedom for the residuals of the model being 381 with 392 as the total sample size, 8 as the conditions and 3 as the number of predictors, covariates included (381 = 392 – 8 – 2 – 1). All values taken from sustained phonation parts is 70 (7 subjects x 5 sustained pitches 2x tasks = 70). df = n-1*

Line 241 has "614,43" which should be "614.43". *🡪 done*
R2.17. Line 249. "right side = task." Should be "right side = task 2."*🡪done*
R2.18. Line 253. This sentence is a bit awkward. *🡪 revised*
R2.19. Line 255. "distances.. These outliners" should be "distances. These outliers". *🡪 done*
R2.20. Line 261. "correlating spectrograph" should be "corresponding spectrogram".*🡪done*
R2.21. Figure 7. Image resolution is poor.*🡪improved to 300dpi*
R2.22. Figures 8 and 9. These are multiple comparisons showing statistically significant differences between measures, but I don't believe any kind of correction (e.g. Bonferroni correction) was used. Some of the significant differences should probably be considered Type I errors. The x-labels are not consistent in both figures.*🡪 We thank the reviewer for this notification – we did the correction via post hoc Turkey-HSD. This information was augmented in the method section. X-Axis label was corrected and standard error was included instead of standard deviation.*

R2.23. Line 380, Line 382, Line 394, and line 468. "adaptions" should be "adaptations". *🡪 done*
R2.24. Line 440. "singiner" should be "singers". *🡪 done*
R2.25. Line 441. "systematical" should be "systematic".*🡪done*
R2.26. Lines 442-444. I don't understand what this sentence is trying to say*.🡪 section was revised*
R2.27. Lines 445-446. I don't understand what this sentence is trying to say. *🡪 section was revised* .
R2.28. Line 471. "interindividually" I think should be deleted. *🡪 done*
R2.29. Line 472. "sex/fach/genre." What is "fach"? *The German* Fach *system is a method of classifying* singers *according to the range, weight, and color of their voices. The word is also used in musical context in English. Section is augmented with details.*
R2.30. Line 477. "patient" should be "patients".*🡪 done*
R2.31. I think could be made more clear at relevant places throughout the manuscript and in Figure 10, what "early" and "late" mean in this paper, i.e. relative to Figure 1*.🡪 we introduced a closer description in the method and again in the result section.*

# Reviewer #3

R3.1: TITLE: I do not believe that the methodology addresses the study of respiratory regu­lation of subglottal pressure because these two measures were estimated in separate sessions. And the data presented by the investigators is on respiratory kinematics given octave pitch jumps.

*We agree that respiratory kinematics is a very good term to describe the data from dynamic MRI measurement. Therefore we included it in the title! Still we believe, that professional singers have very constant breathing patterns which was shown in previous studies (Thomasson & Sundberg, 1999, 2001; Traser et al., 2017). Even if the subglottic pressure measures and MRI were performed in a separate experiment it can be expected that the described kinematics arise for pressure adaptation and that singers used the same subglottic pressure in both parts of the experiment.*

R3.2: ABSTRACT: "During sustained phonation, singers´ diaphragms were elevated and the rip cage diameter was reduced." This sentence appears to state the diaphragm was elevated throughout the sustained phonation. What I believe the authors mean is that the diaphragm continues to elevate in a monotonic manner throughout the sustained phonation. This parti­cular sentence illustrates how the rest of the paper reads and is in need of improvement so the descriptions of data and results are clear.

*We thank the reviewer for the constructive suggestions and revised the manuscript in that manner.*

R3.3: "rip cage" should be changed to "rib cage" throughout.*🡪done*

R3.4: "The magnitude of the movement correlated with the amount of subglottic pressure difference." What is the evidence supporting this statement? I did not see any correlation statistic between diaphragm kinematics and subglottal pressure. Fig. 7 simply displays these variables but I do not see any reference to this highlighted result in the manuscript.

*Indeed, no correlation war calculated here but an univariate ANOVA showed a statistically significant difference in delta of subglottic pressure between high-medium and medium-low jumps. Additionally the mean gradient differed significantly between the high-medium and medium-low jumps. This was clarified throughout the manuscript and the term correlation was avoided in this relation.*

R3.5: INTRODUCTION: "To the author’s knowledge only one attempt has been made using an imaging technique (ultrasound), but this study was limited by the low penetration depth of ultrasound into lung tissue (15)." Even though the cited study had limitations, if it is the only other study investigating parameters of interest, then please much more should be discussed about this reference to put the current work in context. What did the prior work find and how does that lead into the current work?

*More detailed information about this study was included in the introduction and discussion section.*

R3.6: "In a pilot study (22), the movements of the breathing apparatus of 6 professional singers during sustained phonation with a constant ƒo and SPL were analyzed. Here, a very sophisticated movement pattern was observed, which indicated a differentiated control of different parts of the DPH and RC." Please describe more details about the pilot study. These types of statements are good for the introduction but require much more explanation. And please define what is meant by "sophisticated movement pattern."

*More detailed information about the pilot study and the differences to the presented study was included in the introduction section.* The term "sophisticated movement pattern." was also described.

R3.7: The motivation behind the study appears to be in the following paragraph: "Singing is seldom reduced to phonation of a single pitch but characterized by pitch jumps of different magnitude and direction. How different parts of the breathing system move with sudden pitch changes, has not previously been investigated. Thus, the aim of this study was to evaluate the movements of the breathing apparatus during phonation of pitch jumps." However, the motivation that no one else has studied the phenomenon is not adequate. Why do the authors think this study is needed? How can one use the results?

*This section was augmented as suggested.*

R3.8: The hypotheses are inadequate. The current hypotheses are too generic with statements like "respiratory movements differ" and "regulation of pitch jumps would not affect all parts of the respiratory system equally." Please be more specific as to which direction changes are expected and why.

*Hypotheses were elaborated at the end of the introduction section.*

R3.9: METHODS: "At the time of the recording, none of the participants complained of any vocal symptoms or suffered from pulmonary disease (which was confirmed by the VC and FEV1 values in table 1)." This statement mentions subject status at the time of recording. In addition, did any subject have a history of vocal complaints, voice disorders, or respiratory pathologies?

*No subject had any vocal complaints, history of voice disorders, or respiratory pathologies – this is now noted in the manuscript.*

R3.10: Section 2.5 MR Image Analysis: This section on MRI analysis is an important one that defines the primary respiratory kinematic measures of the study. More explanation and detail are necessary than the two sentences currently in this section. Were points on Figure 2 marked manually or with a computer algorithm? If manually, please describe the method used. Who did the ratings? What was their expertise? How many raters marked the images? Multiple raters should be used so measures of reliability can be reported. If algorithmic, please describe the algorithm.

*This section as augmented with more detailed information: Measures were made manually by one examiner (medical doctor, ENT-specialist, with more than 10 years of expertise in analysis of dynamic MRI images) according to the described anatomical landmarks. Repeated measurements of dynamic MRI images of the vocal tract by the same and a second examiner were consistent for all measured articulatory parameters both for the intra rater and inter-rater-reliability in a former study* (Echternach, Burk, Burdumy, Traser, & Richter, 2016b)*.*

R3.11: The term "elevation" of the diaphragm is referred to in the Abstract and Discussion. Please explicitly define how diaphragm elevation was computed. For example, is the DPHhighestP distance what the authors use to define elevation or other measure(s)? Also, "rib cage diameter" is referred to often. Is the rib cage diameter estimated using the apD_Thorax measure or other?

*The DPH elevation consisted of the 3 anatomical distances: anterior, highest point/medium part and posterior DPH (DPHant/med/post). The reduction of the rib cage diameter (apDThorax) is associated with a lowering of the rib cage. These relations are now defined more clearly in the manuscript.*

R3.12: In my opinion, there are too many abbreviations. Please consider spelling out all terms for clarity for the reader. Please go through the manuscript and make sure terms are used consistently and spelled correctly. For example, new abbreviations were used that were not defined in Methods (DPHmed).

*We thank the reviewer for this suggestion. For better readability of the result section we used both the spelled out term and the abbreviation together - abbreviations are needed for the different location measures (for better readability of the figures). DPHmed is now introduced correctly (former DPHhp).*

R3.13: Please put scale bars and orientation directions (anterior, superior, etc.) on Figure 2 and 3.

*We included scale bars and 3D Lung models to show the orientation in figure 2.For Figure 3 we also included descriptions.*

R3.14: "EGG-MRI signal": This signal is undefined. I was initially confused by what this term referred to. Do the authors just mean the EGG signal (recorded during MRI data collection)?

*Yes,* *EGG-MRI signal means the signal that was captured by the EGG electrodes during MRI measurements. This was clarified in the manuscript.*

R3.15: Section 2.5.2: There is an equivalence stated: "t_-4to+4 = m_1 - m_8". This is not an equivalence. Time does not equal distance. And the notation is very loose with a dash (minus sign?) between m_1 and m_8 and "to" between -4 and +4.

*We changed this according to the reviewer’s suggestion - both terms (t_-4_ to t_+4_) and (m_1-8_) are now described in the sentence, not as equivalence.*

R3.16: I highly recommend using the time notation defined (t_n) as x-axis labels in graphs in Fig 6 and 10 so it is clear when jump happens (t0). m1 to m8 are the mean gradients them­selves that should be labeled on the y-axes. Fig 7 is labeled correctly.

*We introduced the label of the x-axis as suggested. We also marked the jump-times.*

R3.17: Anorm is not used again in the manuscript. Is Anorm the y-axis in Fig 7?

*Yes–we introduced Anorm into the label of figure 7*

R3.18: Section 2.6: As mentioned in the abstract comments, the statement in the abstract, "The magnitude of the movement correlated with the amount of subglottic pressure dif­ference," is not supported in the current manuscript. The statistical analysis section does not mention a correlation between respiratory kinematics and subglottal pressure. Please address.

*Indeed, no correlation was calculated here but an univariate ANOVA showed a statistically significant difference in the difference between subglottic pressure valus of two pitches (between high-medium and medium-low jumps). Additionally the mean gradient differed significantly between the high-medium and medium-low jumps. This was clarified throughout the manuscript and the term correlation was avoided in this relation.*

R3.19: Fig 4 subglottal pressure plots: please correct the units on the y-axis to cm H2O.

*Done*

R3.20: Results: In general, while it is appreciated that figure captions include a statement of the result, please revise all caption to better describe/define what each graph, etc., is showing.

*All figure captures were revised for better description.*

R3.20: Section 3.2.1: Fig 6 is another example of wording that needs to be addressed. The text mention tjump, but there is not tjump in Fig 6. As mentioned above, the horizontal axis of Fig 4 should defined in terms of t_n (time).

*According to the reviewers suggestions t_jump_ was included in figure 4 and 6 (now 5 and 7) and the x-axis were labelled with t-4 to t+4*

R3.21: Fig 6 and 7 display mean curves. Please add standard deviation or confidence intervals to provide the reader with an idea of variance across subjects.

*We included standard errors in fig 6 as well as for the subglottic pressure, sound pressure level and open quotient deltas in figure 7. For curves in figure 7 error bars reduced the read­ability a lot. Therefore individual curves are displayed in the supplement figure 1 and 2 to show the variance across subjects.*

R3.22: Fig 8: Graph needs to be corrected for correct spelling of term apDthorax. The incon­sistencies of terms across the manuscript make it very challenging to understand the study. Where is the DPHhighestP measure here? DPHmed here is not defined in Methods.

*DPHhighestP and DPHmed was mixed indeed! We now used the term DPHmed throughout the manuscript and corrected the term everywhere in the manuscript.*

R3.23: Fig 10: Please define "high down," "low down," "high up," and "low up" that is in the le­gend. These terms are not used anywhere else in the manuscript. Please only use terms in the Results section that have already been defined earlier in the manuscript.

*We changed “high”/”low” to the more concise description: “high-medium”/”medium-low” to take the characteristic of the jumps more clear for the reader. These terms were used throughout the manuscript.*

R3.24: "adaption" -> "adaptation"? *Done*
