# Supplementary material for: Respiratory kinematics and the regulation of subglottic pressure for phonation of pitch jumps – a dynamic MRI study
Source: PLoS One. 2020 Dec 31;15(12):e0244539. doi: 10.1371/journal.pone.0244539 (PMC7775092; doi:10.1371/journal.pone.0244539)
Supplement: Attachment — Submitted filename: Response to Reviewers_Revision2_final.docx [file pone.0244539.s007.docx]

# Response to the Reviewers

## Editor Comments

I share the concerns raised by the Reviewers that have to be addressed before acceptance, especially:

- improve english,
- explain and justify performed statistics,
- justify why the authors switch between max and mean values.
- *We have answered these questions carefully, and we hope that the manuscript is now suitable for publication.*

## Reviewer #1

The authors have made considerable revisions and clarifications to the original manuscript. However, while I continue to find the premise, rationale, and data presented in the manuscript to be a valuable contribution to the field my concerns about the validity of the statistical analysis and the clarity of presentation in the manuscript have not been adequately addressed. I therefore cannot recommend this manuscript for publication in its present form.

### Major concerns

1) The author's have not suitably addressed my previous concern that they sometimes analyse maximum measurements and sometimes mean measurements. Section 2.6 attempts to justify this practice, but the meaning of this section is not clear. A direct reading of this section suggests to me that the authors tested for differences between mean and maximum measurements, which would not be a very meaningful thing to do. I suspect that what they have done is to test which measurement gave the largest effects, but this practice is circular and will lead analyses to overestimate effects throughout the paper. The authors need to either provide an a-priori justification for their choices (which it is too late to do for these data), or report all tests and correct appropriately.

- *We thank the reviewer for this comment as it shows that our analysis approach was not clearly explained in the manuscript. We augmented the statistic section in the method part, and the result section:*
  - *Differences in movement pattern occur between upwards and downwards jumps.* *To test whether these differences are depend on the part of the respiratory system (i.e., the anatomical location) two different measures were extracted from the movement curves that optimally describe the characteristics of the movement:*
  - *For downwards jumps (which present with a short inversion of the movement direction) the “maximal gradient” (m_max_) was extracted.* *This value represents the maximum inversion of the movement direction.* *As the maximum value is not meaningful for upwards jumps which are characterized by a steady movement, here the mean gradient (m_mean_) during the jump was used to evaluate whether different location presents a difference in steepness of the gradient over the whole jump window.* *Both values were statistically tested with an univariate ANOVA. Then, statistically significant differences were further analysed with a Turkey’s HSD post-hoc test.*
  - *The following flow chart illustrates the course of the statistical analysis, asso­ciated hypotheses, and figures.*

- ***The choice of maximum vs. mean values is clearly derived from the nature of the motion patterns. It is physically not meaningful to calculate maximum gradients for upward jumps, and/or mean gradients for downward jumps. Therefore, we decided again not to include them in the analysis, as they would not be interpretable.***

2) It is not clear to me that including subject (singer) as a covariate in the ANOVA's is sufficient to solve the problem of non-independence. Repeated measures ANOVA already removes variance between participants to deal with the fact that participants contribute data to each cell of the design. This does not address the problem that in some of the analyses reported, singers contribute multiple data points to the same cell of the design, which as far as I can tell remains a problem in the present manuscript. It should also be point out that the covariate of gender is redundant with the covariate of singer since any variation due to gender will already be accounted for. If it were very important to the authors, singer could be nested with gender do address that question, but I suspect that it would just be a digression.

- *After intensive statistical consultation we conclude that the chosen statistical measure is the best possible option because of the following reasons:*
  - ***We agree with the reviewer, that the best way for a statistical analyzation of the presented data would be to consider only the same jump at each location of each subject differently. This would lead to n=7 which would not allow any statistic at all as the number is too small.***
  - *Including more subjects would be a good solution but professionally trained singers are very rare who are willing to take part in such a demanding study (singing pitch jumps in an MRI machine).*
  - *Another possibility would be to exclude any kind of statistic and simply visually describe the data.*
  - *The method we chose is to pool the data and analyse it with a repeated measures ANOVAs that compares mean values across all variables which are based on repeated observations while controlling for confounding variables (like different subjects, locations and gender) using covariates*
- *In the first version of the manuscript we only included the different subjects and gender as covariates but after the requests of the reviewer we additionally added the different locations as covariates. This changed the values of the statistical parameters, but not their significance levels.*
- ***To make our approach (with all its limitations) more transparent a critical discussion of this part was included in the discussion section:***

*“Additionally, limitations of the statistic evaluation of the presented data must be mentioned. Due to the limited availability of professionally trained singers who are willing to take part in an MRI study and sing pitch jumps in a narrow MR scanner, only 7 subjects were included in this study. Whilst ideally each jump and each location would be analysed individually, a sample size of n=7 did not provide enough data for meaningful statistical analysis in this way. Therefore, our statistical approach included all data in repeated measures ANOVAs with covariates included to test for the effect of confounding variables (like different subjects, locations and gender). This approach was chosen as it allows certain statistical evaluation of the presented data, but the problem of non-independence and heteroscedasticity cannot be solved completely.”*

3) In Section 3.2.1 All measurements are combined in a single test. This may not be the most meaningful way of testing hypothesis A. A value that is smaller than typical for one distance measure may be larger than typical for another. Likewise the various movement locations will have different ranges of values, which I suspect has left a heteroscedasticity problem. The authors should scale the movement directions if they are being combined. Perhaps some kind of multivariate analysis would be more appropriate. A simpler way of testing this hypothesis would be to analyse lung volume (as estimate by the area within the lung in 2d images).

- *We agree with the reviewer, that a heteroscedasticity problem cannot be finally excluded the presented data with the chosen statistic approach – to clarify this limitation to the reader we introduced a limitation section in the discussion section (see answer to point 2)*
- ***We do not agree with the reviewer, that quantification of lung volume from 2D areas would solve the statistical problem.*** ***The advantage of our methodology is that we can make statements on how the individual parts of the respiratory system are regulated in professional singers. In our opinion, this is more valuable then measurements of lung volume which can be acquired more easily by other techniques.***

4) The addition of the derived data sheets to the submission is appreciated. However, the authors should also consider including the raw MRI data. If including the raw audio data would not be consistent with protecting the privacy of participant's then by all means do not include it. Why not provide derived measures from the audio recordings (f0, SPL, etc.) which would then make the raw MRI data useable? I hope that the authors will consider this approach moving forward, as these data would be a useful asset to the field.

- *We made all measured data available for the reader – as supplemental data sheet and as individual movement curves in the supplement figures. The reader can therefor comprehend each individual movement, in each singer and each task.*
- *Additional, to show the quality of the MRI images, an example movie is enclosed, for which we have the permission to be published by the participant.*
- *We do not have the permission by all singers to publish their MR images as a movie even without sound.*
- ***For future studies we will seek the consent of future subjects for a publication of all images (but this comes at the risk that even less subjects are willing to take part of such a study).***

5) Despite many recommendations from all reviewers to improve the writing of the manuscript, there are still a large number of grammatical and spelling errors. In many cases this leads to low intelligibility.

*🡪 The manuscript was checked by a native speaker with special attention on grammar and spelling*

### Minor Concerns

L111 – hypothese & L125 – hypothized *🡪 we changed to “the hypothesis”/ “the* hypotheses*” and “to hypothesise” all over the manuscript*
L125-126 - replace with respectively with i.e.
L282 - pluralise cents, here and throughout
L309 - outliers
L355 - Tukey's-HSD, here and throughout
Many other typographical errors throughout 🡪 *corrected*

## Reviewer #2

The authors have done a good job addressing my comments.

In spite of an acknowledgement for help from a native speaker, there are many grammatical mistakes throughout the revised text, and these will need to be corrected.

- *the manuscript was again checked with special attention on grammar and spelling*

I would recommend the authors to consider referring to their MRI method as "dynamic MRI" rather than "real-time MRI", especially in the title, because "real-time MRI" sounds like rtMRI, which this study does not employ.

- *We thank the reviewer for this comment and changed the title as recommended to dynamic MR*
