# Supplementary material for: Respiratory kinematics and the regulation of subglottic pressure for phonation of pitch jumps – a dynamic MRI study
Source: PLoS One. 2020 Dec 31;15(12):e0244539. doi: 10.1371/journal.pone.0244539 (PMC7775092; doi:10.1371/journal.pone.0244539)
Supplement: Attachment — Submitted filename: Answers to the Reviewer.docx [file pone.0244539.s008.docx]

**Answers to Reviewer #4:**

Reviewer #4: This study uses novel methodology to simultaneously measure movement of multiple structures in the respiratory apparatus relative to pitch jumps during phonation in trained singers. The work should be commended for providing substantial insight into our understanding of how individual components of the respiratory subsystem of phonation contribute to fine biomechanical control and adaptation of voice production. All major limitations of this study have been addressed in previous reviews. Some minor limitations remain, and are listed below:

Abstract:
No comments

Introduction:
1. Lines 118-120 The sentence, "As during sustained phonation the movement range..." is unclear, perhaps because of the complex dependent clause early in the sentence. For ease of reading, the authors might consider the following re-write: "in our pilot study [18], the movement range of the posterior DPH was twice as large as the anterior DPH during sustained phonation. Thus, we additionally hypothesized that there would be differences in psub-adaptive movements of the anterior compared to posterior DPH during pitch jumps.”

- *Changed as suggested*

2. "Pitch jumps" and "octave jumps" are used interchangeably throughout the manuscript. Based on Figure 2, all pitch jumps are octave jumps. The authors should choose either "pitch jumps" or "octave jumps" and use this throughout the manuscript. (ie, Table 1)

*🡪 changed to pitch jumps throughout the manuscript*

Methods
1. The authors do not state whether or not subjects intentionally maintained phonation in a given register (ie falsetto, modal, mixed). Vocal register is still not terribly well understood making discussion challenging, however, a comment on whether register cracks or breaks occurred, whether subjects intentionally maintained a “falsetto” register (which would likely change findings) or completed tasks as they would for performance, or some other mention of the subjects voice quality would facilitate repeatability and allow for a more-nuanced interpretation of findings.

- *We thank the reviewer for hat important suggestion. The subjects were asked to phonate in their western classically trained stage voice without register specification (we included that information in the method section). As you already stated the register terminology is unfortunately until now not standardized. The included professional singers succeeded in their aim to phonate the tasks without unintended register breaks. As the pitch jumps included the whole tessitura of the singers, certainly different registers conditions were used. That a singer used a natural falsetto is unlikely as it can be differentiated from stage voice by the lower sound pressure level and sound quality.* *The influence of register function on the presented data is discussed in a new paragraph in the discussion section.*

2. Line 152: In table 2, subjects 2 and 3 are listed as belonging to Bunch/Chapman taxonomy "3.15b1." However, "3.15" does not exist in the taxonomy. Perhaps the authors meant "3.1b," indicating National/Big City Opera singers singing minor roles?

*🡪 Subjects 2 and 3 are professional choir singers in international touring vocal ensembles.*

*We found that classification – 3.15b1 to be the closest to that*

*From: Bunch M, Chapman J. Taxonomy of singers used as subjects in*

*scientific research. J Voice. 2000;14: 363–9.*

3. Line 161: The shape of the vocal tract will influence glottic configuration and subglottal pressure adaptation. For example, modifications to the vowel /a:/ are often implemented by classically trained singers to facilitate phonation in certain pitch ranges. The authors may wish to comment on whether they feel that subtle shifts in vowel (vocal tract) shape (both within and among subjects) might have influenced the measures of interest in the current study. This would be an appropriate addition to the discussion section.

- *We agree! The vowel [a:] was chosen in all tasks to avoid the articulatory effects that can be expected when fundamental frequency exceeds the normal value of the first format. We discussed the question of interactions between articulation and respiratory movements in a new paragraph in the discussion section together with the question of registers.*

4. Line 174: The authors should comment on the amount of time required for subjects to complete the pitch jumps. If this time was less than 1 second, it is possible that changes in DPH position occurred between samples taken from MRI (ie, might not be detected at 3fps). This would be a helpful addition to the discussion section

*🡪 yes! We included the missing information in the method section and a also a paragraph in the limitation section of the discussion.*

5. Lines 210-217: Measurement of Psub is taken during a task that is different from that performed during MRI. Because the task was not just different in terms of time (ie Psub was not measured during MRI) but also the task itself was different (/pa/ or /pi/ vs /a/), there may have been associated differences in MRI measures of interest. The authors should discuss this when interpreting any results that refer to Psub. In the discussion section.

- *We thank the reviewer for this important comment and included the missing information (phonation of syllable repetition [pa:]) in the method section as well as in the discussion section.*

6. Lines 210-217: Additionally, it is unclear when the /p/ portion of the /p/ occlusion task was performed. (ie, before, during or after the subject reached the target pitch jump?)

*🡪 The syllable [pa:] was repeated 3 times on each pitch, starting the new pitch with a [p:]. Each pitch was held for 3 seconds.*

7. Line 281: In the sentence, "...are possibly influences differently..." it appears that "influences is supposed to be in past tense (ie, "...possibly influenced differently...")

- *Corrected as suggested*

8. Line 291: "Turkey's HSD" should be "Tukey's HSD"

*🡪 Corrected as suggested*

Results:
No Comments

Discussion:
1. The authors may wish to discuss additional ways in which Psub might suddenly change (rather than inspiratory diaphragmatic movement). Ie, is there a way that this might be performed maladaptively at the level of the glottis (ie vocal fold tension) or in the vocal tract (pharyngeal constriction) that would be different between singers and normal controls? Pathological voices vs normal controls?

- *We thank the reviewer for this interesting idea. We included a paragraph in the discussion section. As our primary outcome parameter is the DPH movement we discussed whether this adaptational movement of the DPH to reduce psub is economic. It could be speculated that a failure to reduce the psub by the breathing apparatus could be associated with singing out of tune. However, also glottal, or vocal tract adaptions affect psub. Malregulation of the respiratory system could therefore be related to the necessity of adaptations on glottal or vocal tract level.*

2. Lines 565-583: When "statistic" is used as an adjective, it is typically altered to become “statistical” (ie, "statistical evaluation," as opposed to "statistic evaluation.")

- *Corrected as suggested*
